# Left atrial 4D flow cardiovascular magnetic resonance: a reproducibility study in sinus rhythm and atrial fibrillation

**DOI:** 10.1186/s12968-021-00729-0

**Published:** 2021-03-22

**Authors:** Marco Spartera, Guilherme Pessoa-Amorim, Antonio Stracquadanio, Adam Von Ende, Alison Fletcher, Peter Manley, Stefan Neubauer, Vanessa M. Ferreira, Barbara Casadei, Aaron T. Hess, Rohan S. Wijesurendra

**Affiliations:** 1 Division of Cardiovascular Medicine, Radcliffe Department of Medicine, John Radcliffe Hospital, University of Oxford, West Wing, Headley Way, Oxford, UK; 2 The University of Oxford Centre for Clinical Magnetic Resonance Research (OCMR), Oxford, UK; 3 The University of Oxford Acute Vascular Imaging Centre (AVIC), Oxford, UK; 4 Department of Population Health, CTSU Nuffield University of Oxford, Oxford, UK

**Keywords:** Left atrium, 4D flow, Stasis, Velocities, Vorticity, Vortex, Reproducibility, Cardioembolic risk, Cardiovascular magnetic resonance

## Abstract

**Background:**

Four-dimensional (4D) flow cardiovascular magnetic resonance (CMR) allows sophisticated quantification of left atrial (LA) blood flow, and could yield novel biomarkers of propensity for intra-cardiac thrombus formation and embolic stroke. As reproducibility is critically important to diagnostic performance, we systematically investigated technical and temporal variation of LA 4D flow in atrial fibrillation (AF) and sinus rhythm (SR).

**Methods:**

Eighty-six subjects (SR, n = 64; AF, n = 22) with wide-ranging stroke risk (CHA_2_DS_2_VASc 0–6) underwent LA 4D flow assessment of peak and mean velocity, vorticity, vortex volume, and stasis. Eighty-five (99%) underwent a second acquisition within the same session, and 74 (86%) also returned at 30 (27–35) days for an interval scan. We assessed variability attributable to manual contouring (intra- and inter-observer), and subject repositioning and reacquisition of data, both within the same session (same-day scan–rescan), and over time (interval scan). Within-subject coefficients of variation (CV) and bootstrapped 95% CIs were calculated and compared.

**Results:**

Same-day scan–rescan CVs were 6% for peak velocity, 5% for mean velocity, 7% for vorticity, 9% for vortex volume, and 10% for stasis, and were similar between SR and AF subjects (all p > 0.05). Interval-scan variability was similar to same-day scan–rescan variability for peak velocity, vorticity, and vortex volume (all p > 0.05), and higher for stasis and mean velocity (interval scan CVs of 14% and 8%, respectively, both p < 0.05). Longitudinal changes in heart rate and blood pressure at the interval scan in the same subjects were associated with significantly higher variability for LA stasis (p = 0.024), but not for the remaining flow parameters (all p > 0.05). SR subjects showed significantly greater interval-scan variability than AF patients for mean velocity, vortex volume, and stasis (all p < 0.05), but not peak velocity or vorticity (both p > 0.05).

**Conclusions:**

LA peak velocity and vorticity are the most reproducible and temporally stable novel LA 4D flow biomarkers, and are robust to changes in heart rate, blood pressure, and differences in heart rhythm.

**Supplementary Information:**

The online version contains supplementary material available at 10.1186/s12968-021-00729-0.

## Introduction

Stroke is the second most common cause of death and the third most common cause of disability worldwide [1]. Atrial fibrillation (AF) is a strong risk factor for embolic stroke mainly as it causes pro-thrombotic alterations in atrial blood flow and consequent thrombus formation within the left atrium (LA) [2–5]. Pooled evidence from echocardiography, autopsies, and surgical inspection of the LA reveals that intracardiac thrombus formation generally occurs within the left atrial appendage (LAA), particularly in non-rheumatic heart disease [6, 7]. Overall, as many as 40% of all ischaemic strokes may be due to embolism from the LAA, encompassing not only patients with AF prior to or at the time of the stroke, but also those with AF detected sub-clinically on subsequent cardiac monitoring, as well as a proportion of patients with embolic stroke of unknown origins with signs of atrial myopathy [8].

Prediction of ischaemic stroke risk in clinical practice relies almost exclusively on scoring systems based on upstream clinical risk factors, such as the CHA_2_DS_2_VASc score [9], which apportions one point each for heart failure (C), hypertension (H), age ≥ 65 years (A), age ≥ 75 years (a further point, i.e. A2), diabetes (D), prior thromboembolic disease (S2, two points), vascular disease (VA), and female sex category (Sc). Although higher scores are linked to higher risk of incident ischaemic stroke, this and other similar clinical prediction tools are limited by a modest predictive capacity both in AF and in sinus rhythm (SR) patients [9, 10]. Hence, the ability to assess parameters such as LA blood flow characteristics comprehensively (i.e. absolute flow velocities, the degree of stasis, and vortical flow patterns) both in AF and in SR is very attractive, since these features contribute directly to the downstream pathophysiologic substrate for thrombus formation and may be mechanistically relevant to the risk of embolic stroke [2].

ECG-gated time-resolved phase-contrast cardiovascular magnetic resonance (CMR) with 3-directional velocity encoding (4D flow) is an established CMR technique that allows comprehensive in-vivo visualisation and quantification of cardiovascular flow velocities [11]. Recent proof-of-principle studies have shown that peak and mean velocity and stasis measurements derived from LA 4D flow are associated with clinical stroke risk in patients in SR with history of AF [12, 13] and low blood flow velocities have been linked to activation of the coagulation cascade [2, 3]. Furthermore, assessment of the pattern of blood flow within the LA [14] may also be relevant to embolic risk, since loss of normal vortical flow has been found in patients in SR with a history of paroxysmal AF at higher stroke risk [13] and is associated with platelet aggregation and thrombus formation [15, 16]. Together, these observations raise the intriguing possibility that parameters derived from 4D flow could eventually be used as imaging biomarkers to improve embolic risk stratification in clinical practice, particularly in patients with SR. Nevertheless, it is currently unclear whether LA 4D flow parameters can be measured in a reproducible fashion. Hence, the first step to clinical application is the systematic evaluation of reproducibility and temporal variation, as these directly impact on diagnostic performance and hence the feasibility and power calculations for larger-scale studies. Although analysis of LA 4D flow parameters by CMR is more time-consuming than the collection of simpler measurements, such as LA volume and emptying fraction, direct assessment of a key component of the Virchow’s triad would be highly attractive if able to identify patients who may benefit from anticoagulation, either in SR or with a low AF burden (e.g., not identified by short-term electrocardiographic (ECG) monitoring [17]). In the present study, we aimed to evaluate and compare the reproducibility of a number of LA flow characteristic parameters [12, 13], both within the same session and over time. We included subjects with AF or SR and a wide range of clinical stroke risk, to provide information on reproducibility across a heterogeneous and clinically-relevant population.

## Methods

This study was undertaken in a single tertiary centre (University of Oxford, Radcliffe Department of Medicine, Oxford, UK). The study protocol was approved by a local Research Ethics Committee, and all patients gave written informed consent.

### Design

Patients (in AF or SR) were prospectively non-consecutively recruited from a number of sources at our institution, including outpatient clinics, the waiting list for electrical cardioversion, clinical CMR referrals, a pool of control subjects who had participated in other research studies, and through word of mouth. Young healthy subjects in SR (N = 14, 16%) were recruited under an ethically approved departmental technical development protocol and provided verbal consent (as specified in the protocol).

Subjects were recruited with a wide range of clinical stroke risk, as assessed by the CHA_2_DS_2_VASc score [9]. Patient medical records were screened to document stroke risk factors where available, and a research questionnaire designed to collect this information was administered to all participants.

### CMR imaging protocol

CMR examinations were performed by one of three operators (AF, PM, or MS) on one of two 3T CMR systems (Verio syngo MR B17 and MAGNETOM Prisma VE11C, both Siemens Healthineers, Erlangen, Germany).

At the baseline scan, the CMR protocol included retrospectively ECG-gated time-resolved balanced steady-state free precession (bSSFP) cine imaging in horizontal long axis (‘four-chamber’) and vertical long-axis (‘two-chamber’), for the evaluation of left ventricular (LV) ejection fraction (LVEF) and volumes, and LA emptying function (LAEF) and volumes as previously described [18, 19]. All images were analysed using cvi42 software (version 5.3.4, Circle Cardiovascular Imaging Inc, Calgary, Alberta, Canada).

Additionally, the 4D flow sequence was undertaken twice during the same baseline session with removal of the subject from the scanner and performing repeat isocentre positioning (scan 1a and scan 1b) for determination of ‘same-day scan–rescan’ variability. The same 4D flow sequence was also undertaken again at an interval of approximately 1 month in the same scanner (scan 2) for determination of ‘interval scan–rescan’ variability. In 4 subjects (5%) in whom ‘same-day scan–rescan’ variability had not been determined at scan 1, this was done during scan 2 instead. The 4D flow protocol included retrospectively ECG-gated time-resolved 3D phase-contrast CMR with 3-directional velocity encoding (‘4D flow’) imaging to allow in vivo assessment of blood flow velocities in the LA. In two cases (2%), a prospectively triggered 4D flow sequence was used instead. The 4D flow CMR data were acquired during free breathing using navigator gating of diaphragmatic motion, with pulse sequence parameters as follows: flip angle = 6–7°, spatial resolution/voxel size 2.4 × 2.4 × 2.5-3.0 × 3.0 × 3.0 mm, temporal resolution = 40–49 ms, reconstructed to 20–30 time frames, TE = 2.39–3.66 ms, imaging acceleration (GRAPPA technique) with an acceleration factor of R = 3, total acquisition time = 5–20 min depending on heart rate and navigator efficiency, velocity encoding sensitivity = 110–120 cm/s. The field-of-view (FOV) was axial and adjusted to encompass the entire LA in each subject. Further details of CMR protocols is available in Additional file 1: methods.

### LA 4D flow processing

All 4D flow were acquired with Maxwell term correction [20], DICOM tools from the OXSA toolbox were used [21], and data were corrected for velocity aliasing [22], and eddy currents [23] using a Matlab-based in-house software designed by ATH and MS (MATLAB R2015 8.6.0.267246; Mathworks, Natick, Massachusetts, USA), available on the Oxford University Research Archive (ORA) platform (10.5287/bodleian:ey4ovzdbB). A three-dimensional time-averaged phase contrast angiogram was generated to facilitate better orientation in the context of the LA anatomy and interpretation of the flow data [24]. To isolate the LA velocity data, data analysis included 3D segmentation of the minimum LA volume (directly on magnitude images in the 4D flow dataset) which was obtained using cvi42 software.

Two sets of parameters were derived from 4D flow dataset: (A) absolute velocities and (B) directionality of flow and LA flow patterns.

**Fig. 1:**
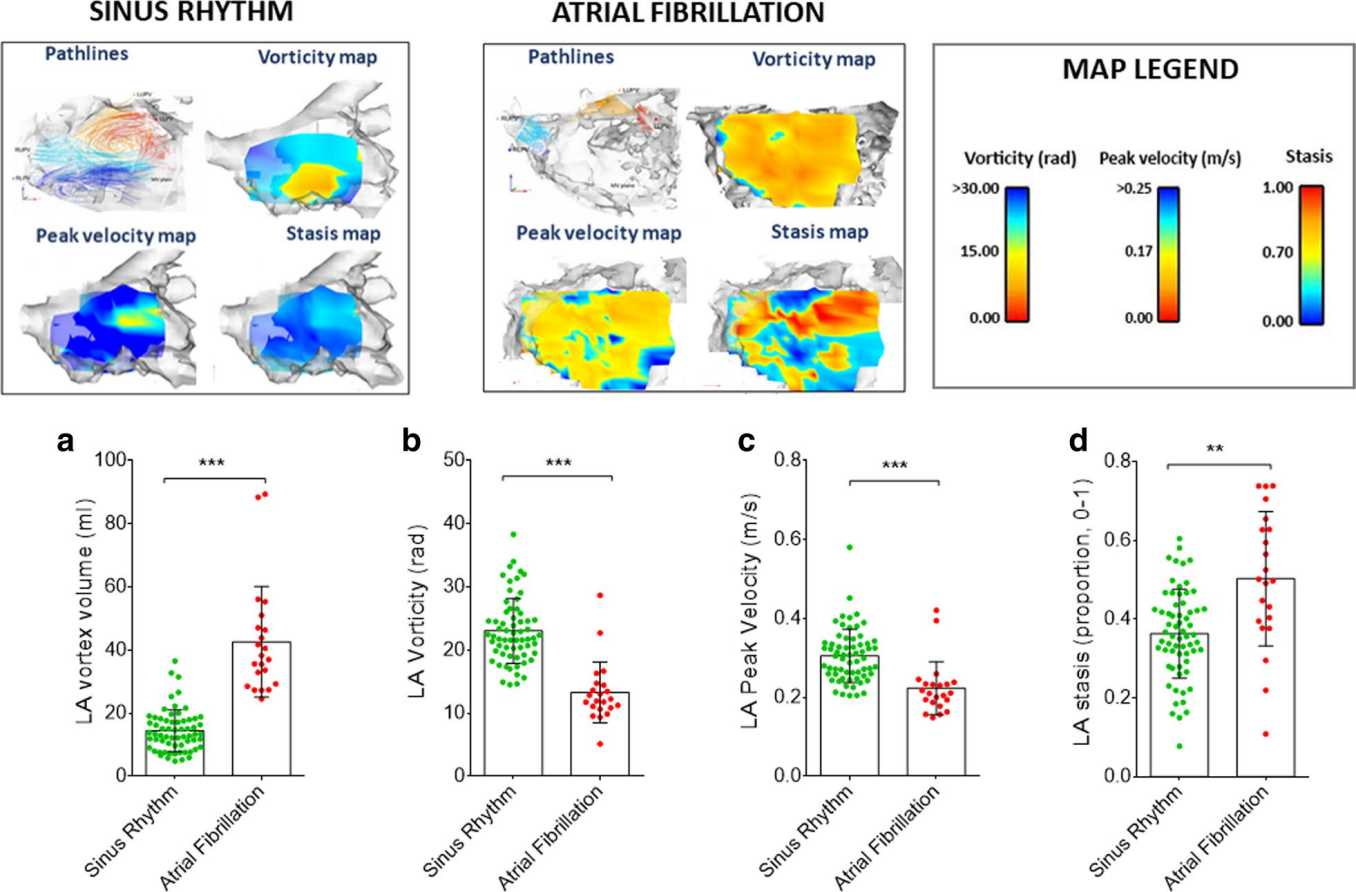
Comparison in left atrial (LA) flow characteristics in sinus rhythm (SR) and atrial fibrillation (AF) at baseline. Upper panel: representative cases of study participants in SR and in AF. Map legends are colour-coded such that hot colours represent deranged 4D flow values. 4D flow imaging in a SR participant (70 year-old male with BMI 30 kg/m^2^ and CHA_2_DS_2_-VASc = 1) shows a typical vortex (see Additional file 2: Video S1 showing pathlines emitted from pulmonary veins). Similar imaging in a patient with AF (67 year-old male with body mass index 26 kg/m^2^ and CHA_2_DS_2_VASc = 3) shows a short-range vortex (see Additional file 2: Video S1). Lower panel: LA mean vortex volume (**a**), LA vorticity (**b**), LA peak velocity (**c**), and LA stasis (**d**) in the overall study cohort stratified per rhythm at the time of the scan. Data expressed as mean ± SD. Statistically significant p values are marked (*p < 0.05), and smaller p values are stratified by size (**p < 0.01, ***p < 0.001). *PV* pulmonary veins, *RU* right upper, *RL* right lower, *LU* left upper, *LL* left lower

The absolute atrial velocities for each voxel inside the segmented LA were determined as described previously [12] (see Fig. 1). Briefly, for each voxel, the peak and mean velocities (m/s) were determined; stasis was calculated as the ratio of the total number of cardiac time frames (n) with a velocity below the threshold of 0.1 m/s (n stasis) to the total number of frames (NTot) [12, 25]: stasis *=*$\frac{{{\text{nstasis}}}}{{{\text{NTot}}}}$. Global LA stasis was defined as the average proportion of stasis across all LA voxels and it is expressed as a proportion from 0 to 1, where 0 is the lowest amount of stasis and 1 is the highest [12, 25].

Regarding flow patterns, a 4D velocity field within the LA was used to generate 3D pathlines and to evaluate atrial flow patterns during both LV systole and diastole using the Runge–Kutta method [26, 27] with Ensight software (2019 R1, ANSYS, Inc., Canonsburg, Pennsylvania, USA), see Fig. 1. Pathlines were emitted at each time-frame from probe planes positioned at each of the pulmonary veins (starting at the beginning of the cardiac cycle), allowing derivation of flow patterns inside the LA during both ventricular systole and diastole (based on mitral valve opening time derived from the magnitude images). Flow patterns were evaluated after quality control steps were applied, as previously described [28]. Normal flow within the LA is characterized by the presence of a vortex [14, 26, 29] (i.e. pathlines with a circular/elliptic trajectory see Fig. 1). Atypical flow patterns observed were classified visually into *short-ranged* vortex (pathlines failing to reach the mitral valve plane during one averaged cardiac cycle, see Fig. 1), and into *other patterns*. Further, for all scans, after flow denoising using divergence free wavelets [30], the volumes of the largest vortex rings inside the LA were calculated automatically using the lambda2 (λ2) method [26]. In each case, a specific lambda threshold for vortices was defined as half the mean λ2 value over the cardiac cycle [13]. LA vortex volume (ml) is reported as the mean of LA vortex volumes throughout the cardiac cycle alongside peak LA vortex volumes (ml) within systole and diastole. Finally, we also applied a measure of tendency of the blood flow to rotate using the volume-averaged magnitude of vorticity (units radians/s) [31, 32], after flow denoising [30]. LA vorticity is reported as the area under the vorticity-time curve over the entire cardiac cycle (in radians or rad), see Fig. 1.

### Blinding

All imaging assessments (including LA and LV function, 4D flow parameters, and flow visualization data) for all the scans (including scan 1a, 1b, and 2) were conducted by investigators blinded to all clinical information including rhythm at the time of the scan. After appropriate training, a single observer (GPA) contoured all cine imaging and LA 4D Flow sequence, according to a dedicated in-house post-processing protocol. A single observer (AS) processed all the qualitative data regarding flow patterns using the Ensight software, according to a separate dedicated in-house post-processing protocol. To evaluate intra-observer variability, the scans were re-contoured by the same observer (GPA) after an interval of at least 2 weeks. Inter-observer variability was conducted independently by a second independent observer (MS) according to the same post-processing protocol, and the 2 observers were blinded to each other. In order to ensure that the observers of scan 1a were blinded to the results of their own analysis of scan 1a and 1b (for intra-/inter- observer CV and rescan CV), the contours performed by GPA/MS were then imported into our MATLAB software (Mathworks) independently by AS who produced the MATLAB output with the results. Finally, to evaluate interval-scan CV, scan 1 and scan 2 were anonymized with a different code in a way that it was impossible to ascertain that scan 1 and 2 belonged to the same research participant.

### Statistical analysis

Data were examined for normality by visual inspection of histograms and Q–Q plots, and for homoscedasticity using Levene’s test. Data are shown as mean ± SD if normally distributed, or median (Q1–Q3) if non-normally distributed, or number (percentage), unless otherwise specified.

To quantify reproducibility and temporal variability, the following metrics were used: Bland-Altman limits of agreement and the within-subject coefficient of variation (CV) [33], which was calculated using the root mean square method and is reported as a proportion (from 0 to 1). Bootstrap resampling (1000 samples) was used to calculate a non-parametric 95% confidence interval (CI) for the CV [33], as well as to estimate 95% CIs and p-values for the difference in CV values between independent groups (i.e. AF vs. SR patients; subjects with longitudinal change ≥ 10 mmHg vs. < 10 mmHg in blood pressure; and subjects with longitudinal change ≥ 10 bpm vs. < 10 bpm in heart rate, where changes are calculated from scan 1a to scan 2). A similar resampling strategy (10,000 bootstrap samples) was used to test differences between dependent CVs (e.g. scan–rescan vs. scan-interval scan). Further, the absolute agreement of the LA flow characteristics values obtained by the two independent observers (i.e. inter-rater variability) was assessed using the intra-class correlation coefficient (ICC) with a two-way mixed model.

Normally-distributed baseline data with equal variances across two independent groups (e.g. AF vs. SR) were compared using the unpaired t-test, and Welch’s tests for normal data with unequal variances. Non-normally distributed unpaired baseline data were compared using the Mann-Whitney U test. Categorical baseline data were compared by using the Chi square or Fischer’s exact tests (if cell size < 5). Flow patterns from related measures were compared with McNemar’s test. Inter-correlations among flow parameters were calculated using Pearson’s coefficient with 1000 bootstraps 95% CI (with sampling stratification for rhythm at the time of the scan).

All tests were 2-tailed, and values of p < 0.05 were considered significant. All p values from pairwise comparisons were adjusted using the Bonferroni method.

We calculated that recruitment of a minimum of 74 subjects would provide 90% power (with two-tailed 0.05 type I error) to detect a 0.01 point mean difference in LA stasis between scan and rescan and between scan and interval scan, with an expected standard deviation of differences of 0.05 and pre-defined clinical agreement limit of 0.15 (based on pilot data).

Statistical analyses were performed with SPSS Statistics for Windows (version 25.0, Statistical Package for the Social Sciences, International Business Machines, Inc., Armonk, New York, USA), R 3.6.3 for computation of CV and bootstrapped 95% CI, GraphPad Prism (version 6.01, GraphPad Software, San Diego, California, USA) for the figures and graphs, and MedCalc Version 19.1.3 (MedCalc Software, Mariakerke, Belgium) for power calculations.

## Results

A total of 86 participants were included: 64 in SR at the time of the CMR scan, and 22 in AF at the time of the CMR scan. Baseline characteristics of all subjects are summarised in Additional file 1: Table S1. As expected, AF patients had profoundly altered LA flow characteristics compared to subjects in SR, as characterised by greater stasis, lower peak velocity, lower vorticity, and larger vortex size (all p ≤ 0.001; Fig. 1), as well as changes in other LA flow measurements (Additional file 1: Table S2). Exploratory analyses assessing LA 4D Flow parameters by age and gender, and their correlation with clinical characteristics are reported in Additional file 1: Tables S3, S4.

A total of 84 baseline scans (SR, n = 62; AF, n = 22) were re-contoured and re-analysed by the same blinded investigator, while 75 baseline scans (SR, n = 53; AF, n = 22) were also re-contoured and re-analysed by a second blinded investigator. A total of 85 participants (SR, n = 63; AF, n = 22) underwent the same 4D Flow sequence twice during the same visit with removal from the scanner and re-positioning for assessment of same-day scan–rescan variability. After an interval of 30 (27–35) days, the scan was repeated (‘scan 2’) in 74 subjects (SR, n = 56; AF, n = 18) for assessment of interval scan–rescan variability. All subjects scanned in SR at scan 1 were in SR at scan 2, and all subjects scanned in AF at scan 1 remained in AF at scan 2.

### Reproducibility and temporal variability in the whole cohort

**Fig. 2:**
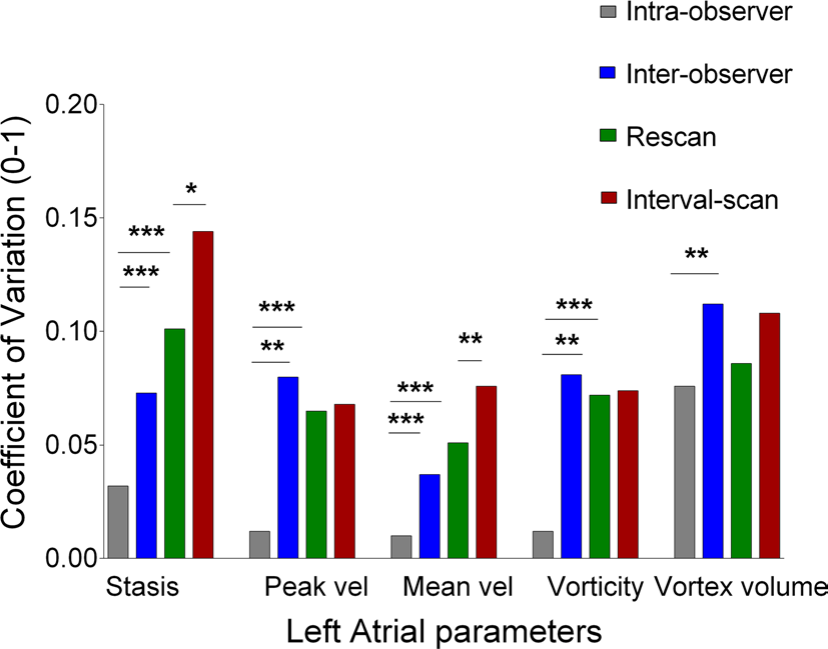
Variability assessment of LA 4D flow parameters. Coefficients of variation (CV) for each flow parameter for intra-observer (grey), inter-observer (blue), rescan (green), and interval scan (red).CV are expressed as proportion (0–1). Statistically significant p values are marked *(< 0.05), and smaller p values are stratified by size (**p < 0.01, ***p < 0.001)

Intra-observer and inter-observer CVs ranged from 1 to 3% and from 4 to 8%, respectively, for LA mean and peak velocity, and stasis (Table 1; Fig. 2), but were larger for vortex volume, which displayed an intra-observer CV of 7.6% and an inter-observer CV of 11.2%. Inter-observer variability was excellent for all parameters (ICC ≥ 0.91, all p < 0.001, Additional file 1: Table S5).

**Table 1:** Reproducibility in the whole cohort

	1	2	3	4				
	Intra-observer N = 84	Inter-observer N = 75	Same-day rescan N = 85	Interval Scan N = 74	p value (1 vs. 2)	p value (1 vs. 3)	p value (2 vs. 3)	p value (3 vs. 4)
LA stasis, %								
CV (95% CI)	0.032 (0.023–0.041)	0.073 (0.052–0.097)	0.101 (0.080–0.124)	0.144 (0.116–0.175)	< *0.001*	< *0.001*	0.076	*0.015*
Bias (BA limits)	0.01 (− 0.02 to 0.02)	0.00 (− 0.09 to 0.07)	0.00 (− 0.09 to 0.09)	− 0.01 (− 0.16 to 0.14)				
LA peak velocity, m/s								
CV (95% CI)	0.012 (0.009–0.015)	0.080 (0.039–0.131)	0.065 (0.042–0.093)	0.068 (0.058–0.079)	*0.007*	< *0.001*	0.645	0.795
Bias (BA limits)	0.00 (− 0.01 to 0.01)	0.01 (− 0.09 to 0.07)	0.00 (− 0.05 to 0.06)	0.00 (− 0.05 to 0.06)				
LA mean velocity, m/s								
CV (95% CI)	0.010 (0.008–0.013)	0.037 (0.025–0.051)	0.051 (0.038–0.064)	0.076 (0.063–0.088)	< *0.001*	< *0.001*	0.134	*0.007*
Bias (BA limits)	0.00 (0.00 to 0.00)	0.00 (− 0.01 to 0.01)	0.00 (− 0.020 to 0.022)	0.00 (− 0.03 to 0.03)				
LA vorticity, rad								
CV (95% CI)	0.012 (0.010–0.014)	0.081 (0.041–0.126)	0.072 (0.052–0.094)	0.074 (0.059–0.090)	*0.002*	< *0.001*	0.816	0.875
Bias (BA limits)	0.00 (− 0.73 to 0.73)	− 0.17 (− 4.67 to 4.32)	− 0.01 (− 4.12 to 4.15)	0.41 (− 3.90 to 4.73)				
LA vortex volume, ml								
CV (95% CI)	0.076 (0.053–0.099)	0.112 (0.083–0.143)	0.086 (0.059–0.115)	0.108 (0.080–0.137)	*0.045*	0.564	0.172	0.254
Bias (BA limits)	− 1.12 (− 5.44 to 3.05)	− 1.18 (− 6.45 to 4.10)	0.42 (− 3.22 to 4.06)	− 0.51 (− 7.32 to 60.30)				

Within-subject coefficients of variation (CV, reported as proportion) are presented with bootstrapped 95% CIs (1000 bootstraps). Mean bias and Bland–Altman (BA) 95% limits of agreement are also reported. p values are obtained by bootstrapped differences (10,000 bootstraps) between CV values for each parameter. Significant p values (< 0.05) are shown in italic

*LA* left atrial

Scan–rescan variability ranged between 5  and 7% for all LA flow parameters, except for vortex volume and stasis (CVs of 9% and 10%, respectively).

Interval-scan variability ranged from 7% (for peak velocity and vorticity) to 14% (for stasis, Table 1; Fig. 2).

Bland-Altman plots are presented in Additional file 1: Fig. S1 (for intra- and inter-observer measurements) and in Additional file 1: Fig. S2 (for rescan and interval scan measurements), with the corresponding mean bias and 95% limits of agreement (LoA) reported in Table 1. Bland–Altman plots revealed no likely proportional bias for any parameter except LA vortex volume which displayed a wider spread at higher values in the interval scan (Table 1 and Additional file 1:  Fig. S2).

Analyses of the reproducibility and temporal variability of qualitative flow patterns assessed by means of pathlines are presented in Table 2. There were no significant paired changes in flow patterns between scan and rescan and no significant changes over time in the interval scans (all p > 0.05; Table 2).

**Table 2:** Reproducibility and temporal variability of flow patterns

	Scan (N = 84)	Same-day rescan (N = 84)	p value	Scan (N = 74)	Interval scan (N = 74)	p value
Systolic patterns						
LA vortex pattern	56 (67)	54 (64)	0.500	50 (68)	51 (69)	> 0.999
LA short-range vortex	18 (21)	19 (24)	> 0.999	16 (22)	15 (20)	> 0.999
Others	10 (12)	11 (13)	> 0.999	8 (11)	8 (11)	> 0.999
Diastolic patterns						
LA vortex pattern	46 (55)	46 (55)	> 0.999	37 (50)	41 (55)	0.454
LA short-range vortex	11 (13)	10 (12)	> 0.999	11 (15)	9 (12)	0.625
Others	27 (31)	28 (33)	> 0.999	26 (35)	24 (32)	0.815

Data presented as N (%). p values refer to paired comparison of proportions by McNemar’s test

### Assessment of sources of variability in LA flow characteristics

In order to assess the sources of variability, comparison among CVs is reported for all flow parameters in Table 1; Fig. 2.

All flow parameters showed statistically significant differences in variability due to manual segmentation by two independent observers (intra-observer vs. inter-observer CV differences from 3 to 7%, all p < 0.05, Table 1; Fig. 2). By contrast, we found no significant differences in variability related to the rescan (i.e., to repositioning and re-acquisition within the same session) over inter-observer variability for any of the parameters evaluated (p > 0.05), although all parameters (except for LA vortex volume) showed a significantly higher rescan variability than intra-observer variability (p < 0.001). Finally, interval-scan variability was significantly higher than the technical rescan variability for LA stasis and LA mean velocity (both p < 0.05), but not for other flow parameters (all p > 0.05) (Table 1; Fig. 2).

Rescan and interval scan CVs, stratified for rhythm at the time of the scan (AF and SR), are shown for each parameter in Additional file 1: Table S6. We found no significant differences in rescan CVs by rhythm for any of the evaluated LA flow parameters (all p > 0.05). However, patients in AF displayed lower interval scan CV for stasis, mean velocity, and vortex volume (all p < 0.05) compared with patients in SR. By contrast, no significant differences in interval scan CVs by rhythm were present for vorticity and peak velocity (all p > 0.05).

Finally, we conducted analysis to investigate whether changes in heart rate (HR) and blood pressure (BP) from scan 1a to scan 2 were associated with differences in interval scan variability (from scan 1a to scan 2) for any of the evaluated LA flow parameters. Interval-scan CVs stratified by ‘ΔHR ≥ 10 bpm vs ΔHR < 10 bpm’ and ‘ΔBP ≥ 10 mmHg vs ΔBP < 10 mmHg’ longitudinal changes are reported for each parameter in Additional file 1:  Tables S7, S8. We found no significant differences in interval-scan CVs by BP for any of the evaluated flow parameters (all p > 0.05). However, ≥ 10 bpm longitudinal changes in HR were associated with higher interval-scan variability for stasis (CV = 17% for ≥ 10 bpm vs. 10% for < 10 bpm; p = 0.024); and were borderline associated with higher interval-scan variability for LA mean velocity (CV = 8.5% for ≥ 10 bpm vs. 6.0% for < 10 bpm; p = 0.067), see Additional file 1: Table S7. Notably, we found that longitudinal changes in HR correlated with changes in LA mean velocity and stasis (p = 0.014 and p = 0.021, respectively), with reduction of HR linked to lower mean velocity and increased stasis (Spearman’s coefficient = − 0.29 and − 0.27, respectively).

Inter-correlation among flow parameters is reported in Additional file 1: Table S9. Peak and mean velocity and stasis all display strong and significant inter-correlation (all r ≥ 0.9, all p < 0.001), whilst vorticity and vortex volume display moderate or mild correlation with the other flow parameters (all r ≤ 0.8, all p < 0.01).

## Discussion

This is the first study investigating reproducibility and temporal variability of LA flow characteristic parameters, which is an important first step to determining their potential clinical applicability and use as imaging biomarkers. Using a large and comprehensive dataset, we quantified the degree of variability for several different flow parameters, and assessed potential contributing sources of errors including manual segmentation, subject positioning in the scanner, data acquisition, temporal changes, and rhythm at the time of the scan. Overall, LA peak velocity and vorticity are the most reproducible, temporally stable, and robust to rhythm differences and heart rate changes over time. Furthermore, these markers provide a non-invasive readout of LA haemodynamics that are potentially mechanistically relevant to thrombus formation and embolic stroke [12, 13].

### Reproducibility and temporal variability

Of the evaluated LA flow parameters, peak and mean velocity and vorticity demonstrated the highest same-day scan–rescan reproducibility, comparable in magnitude to that for other widely-used cardiac imaging biomarkers such as LV end-diastolic volume and LVEF derived from CMR cine imaging, which are reported to have a scan–rescan CV of 5.7% and 6.1%, respectively [33]. These results are also in keeping with prior data on reproducibility of 4D flow assessment of LV blood flow components’ volume ratio, where scan–rescan CVs ranged between 2.5 and 9.2% [34].

As expected, we found an overall incremental variability from intra-observer, to inter-observer and scan–rescan, also in keeping with previous studies [33]. Notably, we found that the variability attributable to human labelling of the LA (intra- and inter-observer CV) was only slightly lower than the scan–rescan variability for LA stasis, and was similar or greater than the scan–rescan variability for all the remaining LA flow parameters. This indicates a high degree of technical reproducibility of LA flow measurement, and is again consistent with CMR assessment of traditional imaging biomarkers such as LVEF [33].

As the ability to detect changes in LA flow parameters over time is of clinical interest, assessment of temporal (scan-interval scan) variability was also a key goal. We found that LA peak velocity and vorticity were the most stable parameters over time, in contrast to stasis and mean velocity, which displayed significantly higher temporal variability compared to their technical variability. Although LA vortex volume was also relatively stable over time, its variability was greater than that of both peak velocity and vorticity.

Importantly, the greater variability recorded with the interval scans over same-day rescan for LA stasis and mean velocity suggests a possible dependence on biological factors. In particular, we found a greater temporal variability in stasis and mean velocity in participants in SR, which was associated with longitudinal changes in heart rate (but not blood pressure). These data are consistent with a previously reported correlation between HR and 4D flow parameters in the LV [34], and are biologically plausible, given that changes in HR affect the dynamics of ventricular filling (i.e. the diastolic phase of the cardiac cycle), and therefore also affect atrial flow components when the mitral valve is open [35–37]. Previous experimental [38] and echocardiographic Doppler studies [37] demonstrated that a higher HR was associated with an increased atrial contribution to ventricular filling by (a) a predominant effect on the atrial flow integral, rather than peak velocities which remained stable [39], and (b) by affecting the passive filling phase, rather than the atrial booster phase [39]. In agreement with previous evidence [39], our data show that peak velocities are stable over time, but we showed for the first time that a reduction in HR is associated with increased LA stasis in SR. If confirmed in larger cohorts, this finding could provide novel insight into the finding that ivabradine (a negatively chronotropic agent) has been associated with an increased risk of incident AF [40] and ischemic stroke in patients with systolic heart failure in SR, in contrast to its beneficial effect on mortality and hospital re-admissions [41]. Nevertheless, HR changes are likely to only partially explain the temporal variability observed in LA stasis, given the mild correlation observed, and other factors are likely to also be involved.

### Suitability for potential clinical application

Low blood flow velocities (linked with activation of the coagulation cascade [2, 3]) and loss of normal coherent vortical flow (linked with platelet aggregation [15, 16]) have been associated with clinical stroke risk in recent validation 4D Flow CMR studies [12, 13]. Further studies assessing the association of LA 4D flow parameters in patients with paroxysmal or undiagnosed AF and thromboembolic events are needed. In order to design and plan such studies, the choice of which LA 4D flow biomarkers are most suitable to be tested for clinical application hinges on (a) reproducibility and temporal variability, (b) selection of those parameters that, in combination, provide complementary/additive information to each other, and (c) feasibility in terms of minimum sample size required for detection of a clinically meaningful difference. .

Of the LA 4D flow parameters evaluated in this study, stasis showed the highest technical and temporal variability, whilst peak velocity and vorticity were the most stable over time. Whilst adjustment for rhythm at the time of the scan is important for all 4D flow parameters to account for the dramatic rhythm-related changes in flow, it is particularly important for stasis, vortex volume, and mean velocity, which displayed higher temporal variability in SR than in AF. Further, the apparent sensitivity of stasis and mean velocity to HR is also relevant, particularly for longitudinal studies, whereas peak velocity and vorticity appear to be heart-rate independent.

Calculations of sample size for future clinical studies are not yet possible due to the lack of large longitudinal studies showing a meaningful significant difference based on prediction of embolic risk or AF. Further research is now needed to identify normal values for these 4D flow parameters in the general population, as well as to determine thresholds that may identify patients at higher risk of incident AF and/or embolic risk.

It is important to emphasise that, in principle, each parameter assesses different aspects of LA flow, and are all therefore potentially relevant markers of the overall propensity for thrombogenesis. Nevertheless, results from our study show that peak and mean velocity and stasis display a significant strong correlation with each other, implying they are likely to convey similar information. On the other hand, vorticity and vortex volume display only moderate or mild inter-correlation with the other parameters, making it more likely that they convey unique and complementary information.

Taken together, our study shows that LA peak velocity and vorticity seem the most promising biomarkers to be tested for potential clinical application, since they are the most reproducible and stable over time, are robust to differences in heart rate and rhythm, and are likely to provide unique information. Both parameters are also mechanistically relevant, since low LA blood flow velocities are linked to activation of the coagulation cascade and increased red blood cell and plasma protein aggregation [2, 3], whilst loss of the normal coherent vortical LA flow pattern [14] is associated with platelet adhesion and aggregation [15, 16].

### Limitations

Firstly, this study was undertaken at a single centre in a mixed cohort with non-consecutive recruitment and reproducibility assessment would need to be confirmed across multiple centres.

Secondly, in the current work, we focussed on the assessment of global LA flow characteristics and did not include the evaluation of LAA flow. Whilst assessment of local flow within the LAA or in other regions of the atrium is possible in principle [12] and clinically relevant, the post-processing tools for this are not widely available, and the reproducibility and temporal variability of LAA flow assessment are unknown. Once a post-processing protocol for LAA flow analysis is available and demonstrated to be reproducible, future studies will be needed to clarify the value of LA analysis compared to LAA analysis, and the relative consequences for clinical applications. Thirdly, although the majority of patients underwent a follow-up scan, analysis of the differences in rescan vs. interval scans variability may be limited by the fact that the subjects who underwent scan 2 are a subset (86%) of those who underwent scan 1. However, the 14% of patients who did not undergo an interval follow-up scan did not display significantly different rescan variability for any parameter compared to those who completed the follow-up visit (all p > 0.05).

In the presented work, we have not compared LA 4D flow parameters against transoesophageal echocardiography as a reference method. Another potential limitation is the use of four different 4D flow protocols (A, B, C, D) and pulse sequences as specified in Additional file 1: methods. We used two similar retrospective protocols (‘A’ and ‘B’, one on each CMR system, both with spatial resolution 3.0 × 3.0 × 3.0 mm and retrospective gating, see further details in Additional file 1: methods) for 90% of the study population. In the remaining 10% of the population, we used 2 different sequences, which were part of our technical development at the beginning of the study: a prospective protocol (‘C’ with the same parameters as protocol ‘A’ but prospectively gated) in 2 participants, and a separate retrospective protocol ‘D’ (with higher spatial resolution: 2.4 × 2.4 × 2.5 mm) in 7 participants (8%). All follow-up scans were performed using the same CMR protocol as the respective baseline scan. We have also reported exploratory analysis of scan–rescan coefficient of variation (CV) for each 4D Flow CMR protocol (available as Additional file 1: Table S10). Overall, the two main retrospective protocols displayed a scan–rescan CV in a similar range to those observed in the overall population.

Finally, 7 subjects (8% of those who underwent an interval scan, 4 in SR and 3 in AF) had changes to prescribed cardiovascular medications at the time of the interval scan compared to baseline. Whilst this could in principle confound the observed changes in LA stasis and mean velocity, there was no evidence of a statistical interaction between changes in these parameters and alterations to medication at follow-up (p = 0.833).

## Conclusions

LA peak velocity and vorticity were the most reproducible and temporally stable novel LA 4D flow biomarkers, and are robust to changes in heart rate, blood pressure, and differences in heart rhythm.

## Supplementary Information

**Additional file 1:** Supplementary material.

**Additional file 2:** Video S1.

## Data Availability

The Matlab-based in-house software designed by ATH and MS (MATLAB R2015 8.6.0.267246; Mathworks, Natick, Massachusetts, USA), has made available on the Oxford University Research Archive (ORA) platform (10.5287/bodleian:ey4ovzdbB). The datasets generated and analysed during the current study are not publically available due to regulatory restrictions. Data that support the findings of this study are available from the corresponding author upon reasonable request.

## Abbreviations

4D flow: Four dimensional flow
AF: Atrial fibrillation
BP: Blood pressure
bSSFP: Balanced steady-state free precession
CI: Confidence interval
CMR: Cardiovascular magnetic resonance
CV: Coefficient of variation
ECG: Electrocardiogram
HR: Heart rate
LA: Left atrium
LAA: Left atrial appendage
LAEF: Left atrial ejection fraction
LoA: Limits of agreement
LV: Left ventricle/left ventricular
LVEF: Left ventricular ejection fraction
ICC: Intra-class correlation coefficient
SD: Standard deviation
SR: Sinus rhythm
