# Supplementary material for: Left atrial 4D flow cardiovascular magnetic resonance: a reproducibility study in sinus rhythm and atrial fibrillation
Source: J Cardiovasc Magn Reson. 2021 Mar 22;23:29. doi: 10.1186/s12968-021-00729-0 (PMC7983287; doi:10.1186/s12968-021-00729-0)
Supplement: Supplementary file 1 — Additional file 1: Supplementary material. [file 12968_2021_729_MOESM1_ESM.docx]

**ADDITIONAL METHODS**

**4D Flow MRI protocols**

CMR examinations were performed on one of two 3 Tesla MRI systems (Verio syngo MR B17 and MAGNETOM Prisma VE11C, both Siemens, Germany). The 4D flow MRI data were acquired during free breathing using navigator gating of diaphragmatic motion, with the following sequence protocols (A, B, C, and D) with breakdown figures of participants that were investigated with each protocol:

| **Protocols** | **Scanner system** | **Acquisition** | **N of participants**  **(% out of 86 total)** |
| --- | --- | --- | --- |
| **Main study protocols** | | | |
| **A** | Verio syngo | Retrospective | 33 (38%) |
| **B** | MAGNETOM Prisma | Retrospective | 44 (51%) |
| **Other research protocols** | | | |
| **C** | Verio syngo | Prospective | 2 (2%) |
| **D** | MAGNETOM Prisma | Retrospective | 7 (8%) |

All follow-up scans were performed using the same MRI protocol as the baseline scan. Protocols details are as follows:

**Main study protocols:**

1. RETROSPECTIVE SEQUENCE - Verio syngo MR B17 Siemens, Germany:

flip angle =6°, spatial resolution/voxel size 3.0×3.0×3.0 mm, temporal resolution (TR) = 48.8 ms, reconstructed to 30 time frames, TE = 3.66 ms, imaging acceleration (GRAPPA technique) with an acceleration factor of R=3, total acquisition time = 10-20 min depending on heart rate, velocity encoding sensitivity = 110 cm/s. The field-of-view (FOV) was axial and adjusted to encompass the entire left atrium in each subject.

1. RETROSPECTIVE SEQUENCE - MAGNETOM Prisma VE11C, Siemens, Germany:

The 4D flow MRI data were acquired during free breathing using navigator gating of diaphragmatic motion, with pulse sequence parameters as follows: flip angle =6°, spatial resolution/voxel size 3.0×3.0×3.0 mm, TR = 43.5 ms, reconstructed to 30 time frames, TE = 3.42 ms, imaging acceleration (GRAPPA technique) with an acceleration factor of R=3, total acquisition time = 5-20 min depending on heart rate, velocity encoding sensitivity = 110 cm/s. The field-of-view (FOV) was axial and adjusted to encompass the entire left atrium in each subject.

**Other research protocols:**

1. PROSPECTIVE SEQUENCE - Verio syngo MR B17 Siemens, Germany:

flip angle =6°, spatial resolution/voxel size 3.0×3.0×3.0 mm, temporal resolution (TR) = 48.8 ms, reconstructed to 14-15 time frames, TE = 3.66 ms, imaging acceleration (GRAPPA technique) with an acceleration factor of R=3, total acquisition time = 5-15 min depending on heart rate, velocity encoding sensitivity = 110 cm/s. The field-of-view (FOV) was axial and adjusted to encompass the entire left atrium in each subject.

1. RETROSPECTIVE SEQUENCE - MAGNETOM Prisma VE11C, Siemens, Germany:

The 4D flow MRI data were acquired during free breathing using navigator gating of diaphragmatic motion, with pulse sequence parameters as follows: flip angle =7°, spatial resolution/voxel size 2.4×2.4×2.5 mm, TR = 39.5 ms, reconstructed to 30 time frames, TE = 2.39 ms, imaging acceleration (GRAPPA technique) with an acceleration factor of R=3, total acquisition time = 10-20 min depending on heart rate, velocity encoding sensitivity = 110 cm/s. The field-of-view (FOV) was axial and adjusted to encompass the entire left atrium in each subject.

**PULSE SEQUENCES USED**

**MAGNETOM Prisma (Protocol B and D)**

These pulse sequences were written in-house by Dr Aaron Hess(1) based on an investigational sequence provided by Siemens. These modifications have now been incorporated by the manufacturer into its investigational sequence, Siemens WIP785. The sequence protocols B and D can therefore be reproduced by other members of the community on VD/VE line scanners with WIP785.

**Verio syngo (Protocol A and C)**

These pulse sequences were written in-house by Dr Aaron Hess and were based on the Siemens WIP785 sequences used for protocols B and D. These sequences will be made available upon request to the authors.

**POST-PROCESSING PROTOCOL**

**Contouring to create 3D mask of the LA:**

3D segmentation is performed in the axial orientation on 4D Flow ‘magnitude images’ derived from the 4D flow dataset with the use of a dedicated CVI42 post-processing software (Short 3D function). Contouring should include all a LA slices between the Pulmonary Artery bifurcation (*top slice)* and the coronary sinus (*bottom slice)*. It is essential to adjust the contours throughout the cardiac cycle so that the contours are consistently within the LA chamber. The resulting 3D mask is exported as *.cvi42wsx file which is subsequently used in MATLAB to isolate the velocity within the LA volume.

**Use of MATLAB software (steps):**

1. Download the software at following the link: <https://doi.org/10.5287/bodleian:ey4ovzdbB> from the ‘Oxford University Research Archive (ORA)’ platform.
2. Copy the “imgradientxyz.m” script from Matlab version R2016a or later to the software folder. The usual path to the “imgradientxyz.m” script is ‘C:\Program Files\MATLAB\Rxxxx\toolbox\images\images’.
3. Export 4D Flow images with DICOM format
4. In the ‘data’ folder of the software, create a new folder containing (1) DICOM file of the 4D Flow images and (2) 3D mask of the LA exported as *.cvi42wsx file. Rename the latter as ‘contours.cvi42wsx’. Take note of the name of the folder and path to the folder.
5. To Select the MRI study to analyse: open ‘Datalist.m’ script and insert the name of the folder where the DICOM file is saved in the “d_path” field;
6. To obtain the list of images within the selected MRI study: open ‘DICOM_LIST_EXTRACTOR.m’ script and fill in the name of the folder in the “evaluate_studies” field.
7. Open ‘Datalist.m’ script and fill in the series numbers corresponding to the 4D Flow magnitude and phase images in the “ser_flow” field.
8. Open ‘run_flow_analysis.m’ script and fill in the name of the folder in the “evaluate_ studies” field.
9. Wait ~10 minutes for the software to calculate all the 4D Flow parameters. You will be able to save the output as .pdf file.

**Visualization of LA flow patterns:**

1. Open Ensight (ANSYS 2019 R1, Canonsburg, PA, USA)
2. Load the .case file (generated after MATLAB processing of 4D Flow dataset)
3. Apply the angiogram generation script (Angiogram.enc) from the command window
4. Place the plane tool at the junction between PVs and LA wall and create a clip plane (i.e. PV tag)
5. Emit twenty-five pathlines (5x5 grid, Emission time start = 0, Total time limit = Full Cycle Duration, Time Delta = time at frame n. 1) from all the PV tags at each time frame. For each case, the time of mitral valve opening (MVO) is used to determine the systolic and diastolic phases
6. Set the view in the –Y plane (coronal view) and animate the generated pathlines

**Time for acquisition and analysis:**

acquisition of 4D Flow sequence may take 5-20 min depending on heart rate and navigator efficiency. After appropriate training, contouring of the LA in cvi42 software takes on average ~5 min. Subsequently, contours are extracted from cvi42 software and loaded in our in-house MATLAB software for calculations (this operation takes ~10 min) to generate the output with quantitative results. Further, after MATLAB processing, the case can be loaded in Ensight software for the visualization of flow patterns (this operation takes on average ~10 min).

**ADDITIONAL TABLES**

**Table S1. Baseline clinical characteristics**

|  |  | Rhythm at the time of scan | | *SR vs AF* |
| --- | --- | --- | --- | --- |
|  | Whole cohort | Sinus Rhythm (SR) | Atrial Fibrillation (AF) | P value |
| **N Total** | **86** | **64** | **22** |  |
| Age, years old | 66 (54 - 71) | 66 (48 - 72) | 66 (58 - 69) | 0.984 |
| Male | 54 (63) | 36 (56) | 18 (82) | **0.041** |
| BMI, Kg/m^2^ | 27.5 ± 5.2 | 27.0 ± 5.4 | 28.8 ± 5.3 | 0.190 |
| BSA, m^2^ | 2.0 ± 0.2 | 1.9 ± 0.2 | 2.1 ± 0.2 | **<0.001** |
| CHA_2_DS_2_VASc score | 2 (0 – 4) | 2 (0 – 4) | 2 (1 – 3) | 0.915 |
| Hypertension | 47 (55) | 34 (53) | 13 (59) | 0.804 |
| Heart Failure | 8 (9) | 2 (3) | 6 (27) | **0.003** |
| Diabetes | 11 (13) | 10 (16) | 1 (5) | 0.276 |
| Previous stroke or TIA | 5 (6) | 3 (5) | 2 (9) | 0.599 |
| Vascular disease | 12 (14) | 9 (14) | 3 (14) | >0.999 |
| Paroxysmal AF | 2 (2) | 2 (3) | 0 (0) | 0.615 |
| Persistent AF | 17 (20) | 0 (0) | 17 (77) | **<0.001** |
| Permanent AF | 5 (6) | 0 (0) | 5 (23) | **0.001** |
| Systolic BP, mmHg | 118 (112 - 128) | 120 (113 - 134) | 113 (109 - 119) | **0.005** |
| Diastolic BP, mmHg | 69 ± 10 | 67 ± 10 | 74 ± 10 | **0.005** |
| Heart Rate, bpm | 67 (60 - 75) | 63 (57 - 69) | 77 (70 - 83) | **<0.001** |
| LV EDV, ml | 150 (127 - 180) | 146 (125 - 170) | 166 (129 - 210) | **0.049** |
| LV EF, % | 60 (50 - 66) | 63 (58 – 67) | 45 (39 - 51) | **<0.001** |
| LA volume, ml | 91 (73 - 116) | 86 (69 - 100) | 120 (101 - 171) | **<0.001** |
| LA EF, % | 44 ± 19 | 54 ± 10 | 17 ± 7 | **<0.001** |
| Values are expressed as mean ± SD or median (q1-q3) or N (%). Significant P values (<0.05) are shown in bold. BMI, body mass index; BSA, body surface area; HR, heart rate; DBP, diastolic blood pressure; SBP, systolic blood pressure; TIA, transient ischaemic attack; LV, Left Ventricle; EDV, End Diastolic Volume; LA, Left Atrium; EF, Ejection Fraction or Emptying Fraction. | | | | |

**Table S2. Baseline LA flow characteristics**

|  | Rhythm at the time of scan | |  |
| --- | --- | --- | --- |
|  | Sinus Rhythm | Atrial Fibrillation | P value |
| **N Total** | **64** | **22** | **-** |
| **Global LA flow** |  |  |  |
| LA stasis, proportion 0-1 | 0.36 ± 0.11 | 0.50 ± 0.17 | **0.001** |
| LA peak velocity, m/s | 0.30 ± 0.07 | 0.22 ± 0.07 | **<0.001** |
| LA mean velocity, m/s | 0.14 ± 0.03 | 0.11 ± 0.03 | **0.001** |
| LA vortex volume (mean), ml | 14 ± 6.6 | 42 ± 17 | **<0.001** |
| LA vorticity, rad | 23.0 ± 5.1 | 13.2 ± 4.8 | **<0.001** |
| **Systolic patterns** |  |  |  |
| LA vortex pattern | 56 (87) | 2 (9) | **<0.001** |
| LA peak vortex volume (systole), ml | 19 ± 9 | 48 ± 22 | **<0.001** |
| LA short-range vortex | 1 (2) | 17 (77) | **<0.001** |
| Other patterns | 7 (11) | 3 (14) | 0.733 |
| **Diastolic patterns** |  |  |  |
| LA vortex pattern | 43 (67) | 5 (23) | **<0.001** |
| LA peak vortex volume (diastole), ml | 18 ± 8 | 48 ± 21 | **<0.001** |
| LA short-range vortex | 0 (0) | 11 (50) | **<0.001** |
| Other patterns | 21 (33) | 6 (27) | 0.629 |
| Values are expressed as mean ± SD or median (q1-q3) or N (%). Significant P values (<0.05) are shown in bold. | | | |

**Table S3. Descriptive statistics for 4D Flow CMR LA parameters stratified for age and gender in sinus rhythm and atrial fibrillation study groups.**

| **SINUS RHYTHM by AGE** | | | | | | |
| --- | --- | --- | --- | --- | --- | --- |
| **N** | **LA 4D Flow parameter** | **Years of age** | **Mean** | **Standard Deviation** | **95% Confidence Interval Lower Bound** | **95% Confidence Interval Upper Bound** |
| 16 | LA stasis, proportion 0-1 | ≤48 | 0.27 | 0.09 | 0.23 | 0.32 |
| 15 |  | 49-66 | 0.38 | 0.09 | 0.33 | 0.44 |
| 17 |  | 67-71 | 0.39 | 0.13 | 0.32 | 0.45 |
| 16 |  | ≥72 | 0.41 | 0.09 | 0.36 | 0.45 |
| 16 | LA peak velocity, m/s | ≤48 | 0.36 | 0.05 | 0.34 | 0.39 |
| 15 |  | 49-66 | 0.29 | 0.05 | 0.26 | 0.32 |
| 17 |  | 67-71 | 0.30 | 0.09 | 0.25 | 0.34 |
| 16 |  | ≥72 | 0.27 | 0.04 | 0.25 | 0.29 |
| 16 | LA mean velocity, m/s | ≤48 | 0.17 | 0.03 | 0.15 | 0.18 |
| 15 |  | 49-66 | 0.14 | 0.02 | 0.12 | 0.15 |
| 17 |  | 67-71 | 0.14 | 0.04 | 0.12 | 0.16 |
| 16 |  | ≥72 | 0.13 | 0.02 | 0.12 | 0.14 |
| 16 | LA vorticity, rad | ≤48 | 28.0 | 4.4 | 25.6 | 30.3 |
| 15 |  | 49-66 | 22.3 | 3.0 | 20.7 | 24.0 |
| 17 |  | 67-71 | 22.5 | 5.6 | 19.6 | 25.4 |
| 16 |  | ≥72 | 19.2 | 2.8 | 17.7 | 20.7 |
| 16 | LA vortex volume (mean), ml | ≤48 | 10 | 4 | 8 | 12 |
| 15 |  | 49-66 | 13 | 6 | 9 | 16 |
| 17 |  | 67-71 | 17 | 7 | 13 | 21 |
| 16 |  | ≥72 | 17 | 7 | 13 | 20 |
| **SINUS RHYTHM by Gender** | | | | | | |
| **N** | **LA 4D Flow parameter** | **Gender** | **Mean** | **Standard Deviation** | **95% Confidence Interval Lower Bound** | **95% Confidence Interval Upper Bound** |
| 36 | LA stasis, proportion 0-1 | Male | 0.36 | 0.11 | 0.33 | 0.40 |
| 28 |  | Female | 0.36 | 0.11 | 0.32 | 0.40 |
| 36 | LA peak velocity, m/s | Male | 0.31 | 0.08 | 0.28 | 0.33 |
| 28 |  | Female | 0.30 | 0.06 | 0.28 | 0.32 |
| 36 | LA mean velocity, m/s | Male | 0.14 | 0.03 | 0.13 | 0.15 |
| 28 |  | Female | 0.14 | 0.03 | 0.13 | 0.15 |
| 36 | LA vorticity, rad | Male | 23.5 | 5.6 | 21.6 | 25.4 |
| 28 |  | Female | 22.4 | 4.5 | 20.7 | 24.2 |
| 36 | LA vortex volume (mean), ml | Male | 17 | 7 | 14 | 19 |
| 28 |  | Female | 11 | 5 | 9 | 13 |
| **ATRIAL FIBRILLATION by Age** | | | | | | |
| **N** | **LA 4D Flow parameter** | **Years of age** | **Mean** | **Standard Deviation** | **95% Confidence Interval Lower Bound** | **95% Confidence Interval Upper Bound** |
| 11 | LA stasis, proportion 0-1 | ≤66 | 0.47 | 0.17 | 0.35 | 0.58 |
| 11 |  | >66 | 0.54 | 0.17 | 0.42 | 0.65 |
| 11 | LA peak velocity, m/s | ≤66 | 0.23 | 0.07 | 0.19 | 0.28 |
| 11 |  | >66 | 0.21 | 0.07 | 0.17 | 0.26 |
| 11 | LA mean velocity, m/s | ≤66 | 0.12 | 0.03 | 0.10 | 0.14 |
| 11 |  | >66 | 0.11 | 0.04 | 0.08 | 0.14 |
| 11 | LA vorticity, rad | ≤66 | 13.9 | 3.6 | 11.5 | 16.3 |
| 11 |  | >66 | 12.6 | 5.9 | 8.6 | 16.5 |
| 11 | LA vortex volume (mean), ml | ≤66 | 42 | 18 | 30 | 54 |
| 11 |  | >66 | 43 | 18 | 31 | 55 |
| **ATRIAL FIBRILLATION by Gender** | | | | | | |
| **N** | **LA 4D Flow parameter** | **Gender** | **Mean** | **Standard Deviation** | **95% Confidence Interval Lower Bound** | **95% Confidence Interval Upper Bound** |
| 18 | LA stasis, proportion 0-1 | Male | 0.51 | 0.19 | 0.42 | 0.60 |
| 4 |  | Female | 0.46 | 0.07 | 0.36 | 0.57 |
| 18 | LA peak velocity, m/s | Male | 0.22 | 0.07 | 0.19 | 0.26 |
| 4 |  | Female | 0.22 | 0.02 | 0.18 | 0.25 |
| 18 | LA mean velocity, m/s | Male | 0.11 | 0.04 | 0.10 | 0.13 |
| 4 |  | Female | 0.12 | 0.01 | 0.10 | 0.13 |
| 18 | LA vorticity, rad | Male | 13.0 | 5.3 | 10.4 | 15.6 |
| 4 |  | Female | 14.3 | 1.9 | 11.3 | 17.3 |
| 18 | LA vortex volume (mean), ml | Male | 45 | 19 | 35 | 54 |
| 4 |  | Female | 33 | 8 | 21 | 45 |
| Exploratory descriptive statistics of each LA parameter by 4D Flow CMR are reported with stratification by age and gender in sinus rhythm and atrial fibrillation study groups. In sinus rhythm, age brackets follow the quartiles of age distributions. In atrial fibrillation, age brackets are based on values below and above the median of age distribution. | | | | | | |

**Table S4. Correlation between LA flow parameters and clinical parameters**

| (N=86)  **4D Flow Parameters** | **Age** | **Body mass index** | **Body surface area** | **CHA2DS2VASc score** |
| --- | --- | --- | --- | --- |
| **LA stasis** | 0.32** | -0.047 | 0.14 | 0.20 |
| **LA peak velocity** | -0.34** | -0.12 | 0.05 | -0.29** |
| **LA mean velocity** | -0.34** | -0.03 | -0.02 | -0.25* |
| **LA vorticity** | -0.35** | -0.13 | -0.23* | -0.19 |
| **LA vortex volume** | 0.26* | 0.08 | 0.42** | 0.17 |
| Spearman’s Correlation Coefficients were calculated. Significant P values are stratified as follows: * (P<0.05), ** (P<0.01). | | | | |

**Table S5. Inter-rater Correlation Coefficient (ICC).**

|  | Inter-rater ICC (95% CI)  **N = 75** | p value |
| --- | --- | --- |
| LA stasis, proportion 0-1 | 0.98 (0.96 – 0.98) | **p < 0.001** |
| LA peak velocity, m/s | 0.91 (0.85 – 0.94) | **p < 0.001** |
| LA mean velocity, m/s | 0.99 (0.98 – 0.99) | **p < 0.001** |
| LA vorticity, rad | 0.97 (0.95 – 0.98) | **p < 0.001** |
| LA vortex volume, ml | 0.99 (0.98 – 0.99) | **p < 0.001** |
| Inter-Rater Correlation Coefficient (ICC) is reported with (95% CI). Significant P values (<0.05) of the two-way mixed effects models are shown in bold. LA; Left Atrium. | | |

**Table S6. Coefficient of Variations for rescan and interval-scan variability by rhythm at the time of the scan.**

|  | **SR** | **AF** | **Difference**  **(AF-SR)** | **Lower 95%CI** | **Upper 95%CI** | **P value** |
| --- | --- | --- | --- | --- | --- | --- |
| **CV scan-rescan** |  |  |  |  |  |  |
| LA stasis | 0.104 | 0.085 | -0.019 | -0.07 | 0.039 | 0.504 |
| LA mean velocity | 0.049 | 0.054 | 0.006 | -0.025 | 0.042 | 0.759 |
| LA peak velocity | 0.051 | 0.089 | 0.038 | -0.014 | 0.105 | 0.271 |
| LA vortex volume | 0.093 | 0.055 | -0.038 | -0.077 | -0.002 | 0.051 |
| LA vorticity | 0.057 | 0.103 | 0.046 | -0.003 | 0.094 | 0.062 |
| **CV scan-interval** |  |  |  |  |  |  |
| LA stasis | 0.161 | 0.075 | -0.087 | -0.128 | -0.046 | **<0.001** |
| LA mean velocity | 0.082 | 0.055 | -0.027 | -0.048 | -0.006 | **0.013** |
| LA peak velocity | 0.07 | 0.064 | -0.006 | -0.026 | 0.013 | 0.546 |
| LA vortex volume | 0.119 | 0.063 | -0.056 | -0.094 | -0.018 | **0.004** |
| LA vorticity | 0.069 | 0.085 | 0.016 | -0.018 | 0.048 | 0.353 |
| CV; Coefficient of Variation. CI; Confidence Interval. LA; Left Atrium. Significant P values (<0.05) are shown in bold. | | | | | | |

**Table S7. Coefficient of Variations for interval-scan variability by heart rate paired changes between baseline and interval-scan.**

|  | **Group A (N=49)** | **Group B (N=24)** | **Difference**  **(group B- group A)** | **Lower 95% CI** | **Upper 95% CI** | **P value** |  |
| --- | --- | --- | --- | --- | --- | --- | --- |
|  | HR longitudinal change <10 bpm | HR longitudinal change ≥10 bpm |  |  |  |  |  |
| **CV Scan-interval** |  |  |  |  |  |  |  |
| LA stasis | 0.104 | 0.173 | 0.069 | 0.008 | 0.125 | **0.024** |  |
| LA mean velocity | 0.060 | 0.085 | 0.025 | -0.002 | 0.051 | 0.067 |  |
| LA peak velocity | 0.061 | 0.068 | 0.006 | -0.016 | 0.028 | 0.605 |  |
| LA vortex vol | 0.088 | 0.119 | 0.031 | -0.029 | 0.085 | 0.330 |  |
| LA vorticity | 0.065 | 0.076 | 0.010 | -0.020 | 0.038 | 0.497 |  |
| CV; Coefficient of Variation. CI; Confidence Interval. Heart Rate; HR. LA; Left Atrium. Significant P values (<0.05) are shown in bold. | | | | | | | |

**Table S8. Coefficient of Variations for interval-scan variability by blood pressure paired changes between baseline and interval-scan.**

|  | **Group A**  **(N=45)** | **Group B**  **(N=28)** | **Difference**  **(group B- group A)** | **Lower 95% CI** | **Upper 95% CI** | **P value** |  |
| --- | --- | --- | --- | --- | --- | --- | --- |
|  | Systolic BP longitudinal change <10 mmHg | Systolic BP longitudinal change ≥10 mmHg |  |  |  |  |  |
| **CV scan-interval** |  |  |  |  |  |  |  |
| LA stasis | 0.130 | 0.140 | 0.010 | -0.048 | 0.067 | 0.723 |  |
| LA mean velocity | 0.066 | 0.077 | 0.011 | -0.014 | 0.037 | 0.412 |  |
| LA peak velocity | 0.058 | 0.071 | 0.013 | -0.008 | 0.033 | 0.228 |  |
| LA vortex vol | 0.087 | 0.118 | 0.031 | -0.024 | 0.083 | 0.287 |  |
| LA vorticity | 0.068 | 0.072 | 0.004 | -0.026 | 0.034 | 0.840 |  |
|  | **Group C**  **(N=59)** | **Group D**  **(N=14)** | **Difference**  **(group D- group C)** | **Lower 95% CI** | **Upper 95% CI** | **P value** |  |
|  | Diastolic BP longitudinal change <10 mmHg | Diastolic BP longitudinal change ≥10 mmHg |  |  |  |  |  |
| **CV scan-interval** | 0.145 | 0.099 | -0.046 | -0.116 | 0.020 | 0.164 |  |
| LA stasis | 0.075 | 0.057 | -0.019 | -0.055 | 0.016 | 0.269 |  |
| LA mean velocity | 0.068 | 0.053 | -0.014 | -0.045 | 0.013 | 0.290 |  |
| LA peak velocity | 0.106 | 0.085 | -0.021 | -0.067 | 0.024 | 0.370 |  |
| LA vortex vol | 0.071 | 0.065 | -0.006 | -0.049 | 0.036 | 0.693 |  |
| LA vorticity | 0.145 | 0.099 | -0.046 | -0.116 | 0.02 | 0.164 |  |
| CV; Coefficient of Variation. CI; Confidence Interval. BP; Blood Pressure. LA; Left Atrium. Significant P values (<0.05) are shown in bold. | | | | | | | |

**Table S9. Inter-correlation among LA flow parameters stratified by rhythm**

|  | **LA stasis** |  |  |  |
| --- | --- | --- | --- | --- |
| **LA peak velocity** | -0.88**  (-0.84 to -0.92) | **LA peak velocity** |  |  |
| **LA mean velocity** | -0.94**  (-0.96 to -0.93) | 0.95**  (0.92 to 0.97) | **LA mean velocity** |  |
| **LA vorticity** | -0.71**  (-0.84 to -0.92) | 0.81**  (0.71 to 0.89) | 0.75**  (0.64 to 0.83) | **LA vorticity** |
| **LA vortex volume** | 0.34**  (0.02 to 0.71) | -0.38**  (-0.70 to -0.13) | -0.26*  (-0.66 to 0.07) | -0.47**  (-0.71 to -0.24) |
| Pearson’s Correlation Coefficients were calculated with bootstrapped 95%CI (1000 resamples) stratified by rhythm. Significant P values are stratified as follows: * (P<0.05), ** (P<0.01). | | | | |

**Table S10. Scan-rescan coefficient of variation for left atrial 4D Flow parameters according to each CMR protocol**

|  |  | **Main protocols** | | **Others** | |
| --- | --- | --- | --- | --- | --- |
| **(% of study population)** | **Overall** | Protocol A  (**38%**) | Protocol B  (**51%**) | Protocol C  (**2%**) | Protocol D  (**8%**) |
| Stasis | 0.101 | 0.098 | 0.082 | 0.241 | 0.149 |
| Peak velocity | 0.065 | 0.054 | 0.048 | 0.273 | 0.063 |
| Mean velocity | 0.051 | 0.047 | 0.036 | 0.153 | 0.084 |
| Vorticity | 0.072 | 0.073 | 0.051 | 0.203 | 0.110 |
| Vortex vol | 0.086 | 0.068 | 0.072 | 0.041 | 0.194 |

**Figure legend**

Figure S1. Bland-Altman plot of intra-observer (A, C, E, G, I) and inter-observer (B, D, F, H, J) variability in LA stasis, LA peak velocity, LA mean velocity, LA vorticity, and LA vortex volume in the whole population.

**Figure S2.** Bland-Altman plot of rescan variability **(A, C, E, G, I**) and interval scan variability (**B, D, F, H, J**) in LA stasis, LA peak velocity, LA mean velocity, LA vorticity, and LA vortex volume in the whole population.

**Figure S1**

**Figure S2**

1. Stoll VM, Loudon M, Eriksson J et al. Test-retest variability of left ventricular 4D flow cardiovascular magnetic resonance measurements in healthy subjects. J Cardiovasc Magn Reson 2018;20:15.
